# Clinical analysis of adrenal lesions larger than 5 cm in diameter (an analysis of 251 cases)

**DOI:** 10.1186/s12957-019-1765-7

**Published:** 2019-12-16

**Authors:** Zongzong Zhang, Lina Wang, Jing Chen, Xiunan Li, Dikuan Liu, Tianyu Cao, Xuehan Yang, Hongwei Huang, Xuejian Wang, Xishuang Song, Deyong Yang, Jianbo Wang

**Affiliations:** 1 Department of Urology, The First Affiliated Hospital of Dalian Medical University, Dalian, China; 2 Department of Radiology, The First Affiliated Hospital of Dalian Medical University, Dalian, China

**Keywords:** Large adrenal tumors, Adrenalectomy

## Abstract

**Background:**

To describe the pathological distribution, imaging manifestations, and surgical managements and prognosis of large adrenal tumors (LATs) ≥ 5 cm

**Methods:**

A total of 251 patients with LATs were analyzed on the basis of pathological or clinical diagnosis. Regarding surgery, open adrenalectomy was performed on 89 patients, and laparoscopic adrenalectomy was performed on 89 patients. Thirty-two patients with bilateral tumors were analyzed in terms of clinical characteristics. The survival rate was determined for 43 patients with adrenal metastases and 29 patients with primary adrenal malignancies. The CT characteristics including tumor diameter, shape, edge, heterogeneity, necrosis, calcification, pre-contrast attenuation, and contrast attenuation were analyzed for 117 patients.

**Results:**

The majority of LATs were still benign, but they had a higher probability to be malignant. Benign LATs made up 68.13% of all cases, mainly adrenal cysts (19.52%), pheochromocytoma (18.73%), benign adenoma (16.73%), and myelolipoma (7.17%). Malignant LATs accounted for 28.69% of cases, mainly including adrenocortical carcinoma (8.76%) and metastases (17.13%). Laparoscopic surgery was found to involve less trauma than open surgery. It was also safer and postoperative recovery was faster, but it had drawbacks and could not completely replace open surgery. CT features had obvious specificity for the diagnosis of benign and malignant tumors. For example, benign adenomas had a smaller pre-contrast (< 10 Hu) whereas malignant adrenal tumors had, on the contrary, higher attenuation. Regarding adrenal malignant carcinoma, adrenal primary malignant tumors showed a better prognosis than adrenal metastases (mean survival of 19.17 months vs 9.49 months). Primary adrenal cortical carcinoma without metastasis had a better prognosis than primary adrenal cortical carcinoma metastasis (mean survival of 23.71 months vs 12.75 months), and adrenal solitary metastasis had a better prognosis than general multiple metastatic carcinoma (mean survival of 14.95 months vs 5.17 months).

**Conclusion:**

LATs were more likely to be benign; however, they still had a high probability of being a malignant tumor. Understanding the clinicopathological characteristics of LATs can facilitate selection of more effective clinical treatment options.

## Background

With the development of imaging technology, there is an increasing number of adrenal incidentalomas discovered incidentally. However, large adrenal tumors (LATs) are considered uncommon, with an incidence ranging from 8.6 to 38.6% [1, 2]. The possibility of malignancy increases with the increasing tumor volume [3–6]. For LATs, accurate preoperative assessments of the nature and origin of the tumors are important to the choice of therapy [7–9].

By definition, LATs range in size from more than 5 to 10 cm in diameter, with a consensus of approximately 5 cm [3, 10]. LATs cover a spectrum of various pathologies, ranging from benign lesions to adrenocortical carcinoma or metastasis. Among 19 histopathological diagnoses, the most common was found to be adrenal cancer, followed by benign adenomas, pheochromocytomas, and metastasis [3]. The prognosis of different pathological types of LATs was found to differ. Patients with malignant tumors exhibited poor prognosis, especially those with adrenal metastasis [11]. Accurate diagnosis and functional evaluation of LATs and preoperative differentiation of benign and malignant tumors have great significance for the selection of appropriate treatment approaches. Functional adrenal tumors and local invading lesions require more preoperative preparation [3] and may even affect the choice of surgical approach [7]. Because the possibility of an uncomplete curative resection with the laparoscopic adrenalectomy, open adrenalectomy is indicated in some cases [12]. The clinical, imaging, and pathological features of LATs play an important role in preoperative judgment of the nature of each tumor and selection of the best treatment.

In the present study, the clinical data of 251 patients with LATs in our hospital during a 10-year period from 2009 to 2018, including the pathological distribution, imaging manifestations, and surgical managements and prognosis, were collected and analyzed to provide a basis for the treatment of LATs.

## Methods

From January 2009 to August 2018, among the patients admitted to the First Affiliated Hospital of Dalian Medical University, 1160 showed adrenal tumors in imaging reports. In this group, a total of 251 patients who had imaging manifestations showing that the size of adrenal tumors was larger than or equal to 5 cm were identified. Patient information (age, sex, survival time), laboratory work-up (blood cortisol, blood aldosterone, ACTH), tumor character (pathological diagnosis, imaging manifestations, lesion location, diameter), and information related to the operation (surgical methods, bleeding volume, blood transfusion volume, operation time, drainage time, hospitalization time) were collected. All patients with a preoperative diagnosis of pheochromocytoma received specific preparations to control hypertension and facilitate rehydration. Most of these preparations included α receptor blockers and equilibrium salt solution.

Among these patients, 178 underwent urological surgery. The typical surgical management plans were laparoscopic adrenalectomy and open adrenalectomy. Eighty-nine patients underwent open adrenalectomy. Laparoscopic adrenalectomy was performed in 89 patients. Seventy-three patients did not undergo surgery, and clinical doctors made clinical diagnoses based on thorough history, clinical evaluation, hormonal and biochemical workup, and imaging reports.

The survival rates of 43 patients with adrenal metastasis and 29 patients with primary adrenal malignancies were determined according to the follow-up by phone. Two-month, 6-month, 12-month, 24-month, and 30-month survival rates were analyzed.

CT images of 117 patients with benign adenoma, pheochromocytoma, cortical carcinoma, metastatic carcinoma, and gangliocytoma were collected. The CT characteristics including tumor diameter, shape, edge, heterogeneity, necrosis, calcification, pre-contrast attenuation, and contrast attenuation of the 117 patients were analyzed.

### Statistical analysis

SPSS 21.0 software was used for statistical analysis. In case of skewed distribution and categorical data, rank sum test was used. A *P* value of < 0.05 was considered statistically significant for all tests.

## Results

The pathological distribution of LATs and the characteristics of patients are shown in Table 1. Tumor size measured on histopathological examination or imaging measurement ranges from 50 to 200 mm. The five most common LATs, including adrenal cyst (19.52%), pheochromocytoma (18.73%), metastases (17.13%), benign adenoma (16.73%), and adrenal cortical cancer (8.76%), were responsible for 80.88% of cases in this study. Benign LATs, including adrenal cyst (19.52%), pheochromocytoma (18.73%), benign adenoma (16.73%), myelolipoma (7.17%), gangliocytoma (3.19%), schwannoma (0.8%), teratoma (1.20%), and hemangioma (0.80%), were responsible for 68.13%. Malignant LATs, mainly including metastases (17.13%), cortical carcinoma (8.76%), small cell carcinoma (1.20%), lymphoma (1.20%), and melanoma (0.4%), accounted for 28.69% of all cases.

**Table 1:** Clinical features of adrenal tumors larger than 5 cm in diameter

Diagnosis	Number, *n* = 251	Age (years) (mean ± SD)	Male/female	Diameter (cm) (mean ± SD)	Location right/left
Adrenal cyst	49 (19.52%)	42.51 ± 14.28	21/28	8.19 ± 2.97	18/31
Pheochromocytoma	47 (18.73%)	50.21 ± 15.02	24/23	7.46 ± 2.46	21/26
Benign adenoma	42 (16.73%)	58.24 ± 13.64	20/22	6.38 ± 1.67	21/21
Myelolipoma	18 (7.17%)	56.22 ± 11.39	6/12	8.58 ± 2.03	16/2
Gangliocytoma	8 (3.19%)	41.13 ± 13.74	6/2	7.85 ± 2.73	5/3
Schwannoma	2 (0.80%)	57.50 ± 8.50	0/2	8.55 ± 3.05	1/1
Teratoma	3 (1.20%)	49.00 ± 12.57	0/3	9.30 ± 1.38	2/1
Hemangioma	2 (0.80%)	42.50 ± 14.50	0/2	9.37 ± 2.87	2/0
Cortical carcinoma	22 (8.76%)	55.05 ± 15.89	9/13	8.97 ± 3.33	13/9
Small cell carcinoma	3 (1.20%)	41.33 ± 8.65	2/1	9.90 ± 5.77	1/2
Lymphoma	3 (1.20%)	66.67 ± 10.40	0/3	6.48 ± 0.76	0/3
Melanoma	1 (0.40%)	54	0/1	9.5	0/1
Metastase	43 (17.13%)	63.91 ± 11.16	34/9	7.64 ± 2.99	24/19
Undefined	8 (3.19%)	57.25 ± 12.10	4/4	7.85 ± 3.88	2/6

Some obvious features in certain types of LATs were observed in the present study. The mean age of patients with lymphoma (66.67 years) and metastases (63.91 years) was higher than in other groups. In the case of metastases, the male/female ratio was 34/9, with a higher proportion of males than other groups. In patients with adrenal myelolipoma, most of the lesions were on the right side (R/L = 16:2).

Patients and tumor characteristics of bilateral LATs are shown in Table 2. Among the bilateral LATs, the most common diagnostic was malignant tumor (71.88%), followed by benign adenoma (15.63%), myelolipoma (6.25%), gangliocytoma (3.13%), and pheochromocytoma (3.13%). The most common type of malignant tumor was metastatic (50%), followed by cortical carcinoma (15.63%) and lymphoma (6.25%). Among adrenal metastases, lung cancer was the main source, accounting for 50% of all adrenal metastases. It was followed by kidney cancer (12.5%), intestinal malignancy (12.5%), and prostate cancer (6.26%).

**Table 2:** Characteristics of bilateral adrenal tumors larger than 50 mm

Diagnosis	Number *n* = 32	Age (years) (mean ± SD)	Male/female	diameter (cm) (mean ± SD)	
				R	L
Benign adenoma	5 (15.63%)	64.80 ± 9.24	5/0	2.64 ± 2.04	5.72 ± 2.96
Cortical carcinoma	5 (15.63%)	52.40 ± 12.18	3/2	8.95 ± 3.12	5.49 ± 4.51
Myelolipoma	2 (6.25%)	53.50 ± 6.50	1/1	7.85 ± 0.55	1.40 ± 0.60
Lymphoma	2 (6.25%)	74 ± 1	0/2	4.02 ± 1.49	6.93 ± 0.53
Gangliocytoma	1 (3.13%)	54	1/0	7.50	4.00
Pheochromocytoma	1 (3.13%)	66	0/1	6.50	0.60
Metastatic carcinoma	(50.00%)				
Lung cancer	8 (25.00%)	62.63 ± 9.14	6/2	4.85 ± 3.68	4.02 ± 1.94
Kidney cancer	2 (6.25%)	61.50 ± 1.50	2/0	4.90 ± 0.70	5.15 ± 3.35
Intestinal malignancy	2 (6.25%)	64.50 ± 0.50	2/0	2.25 ± 0.75	6.83 ± 0.17
Prostate cancer	1 (3.13%)	80	1/0	1.80	6.05
Urothelial carcinoma	1 (3.13%)	84	1/0	10.73	4.00
Liver cancer	1 (3.13%)	63	1/0	5.53	5.00
Melanoma	1 (3.13%)	45	1/0	1.95	15.16

**Fig. 1:**
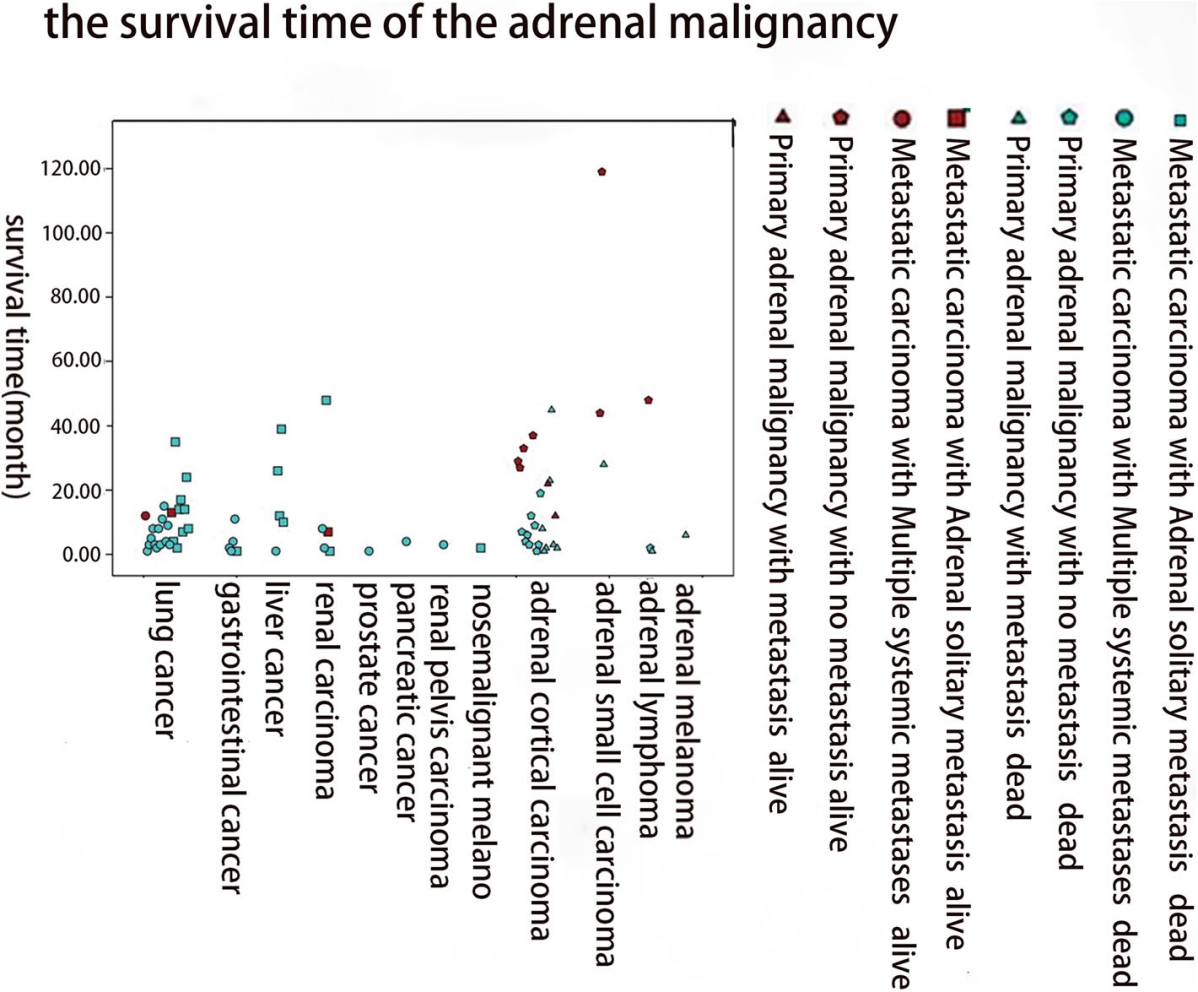
The survival time of the adrenal malignancy

**Fig. 2:**
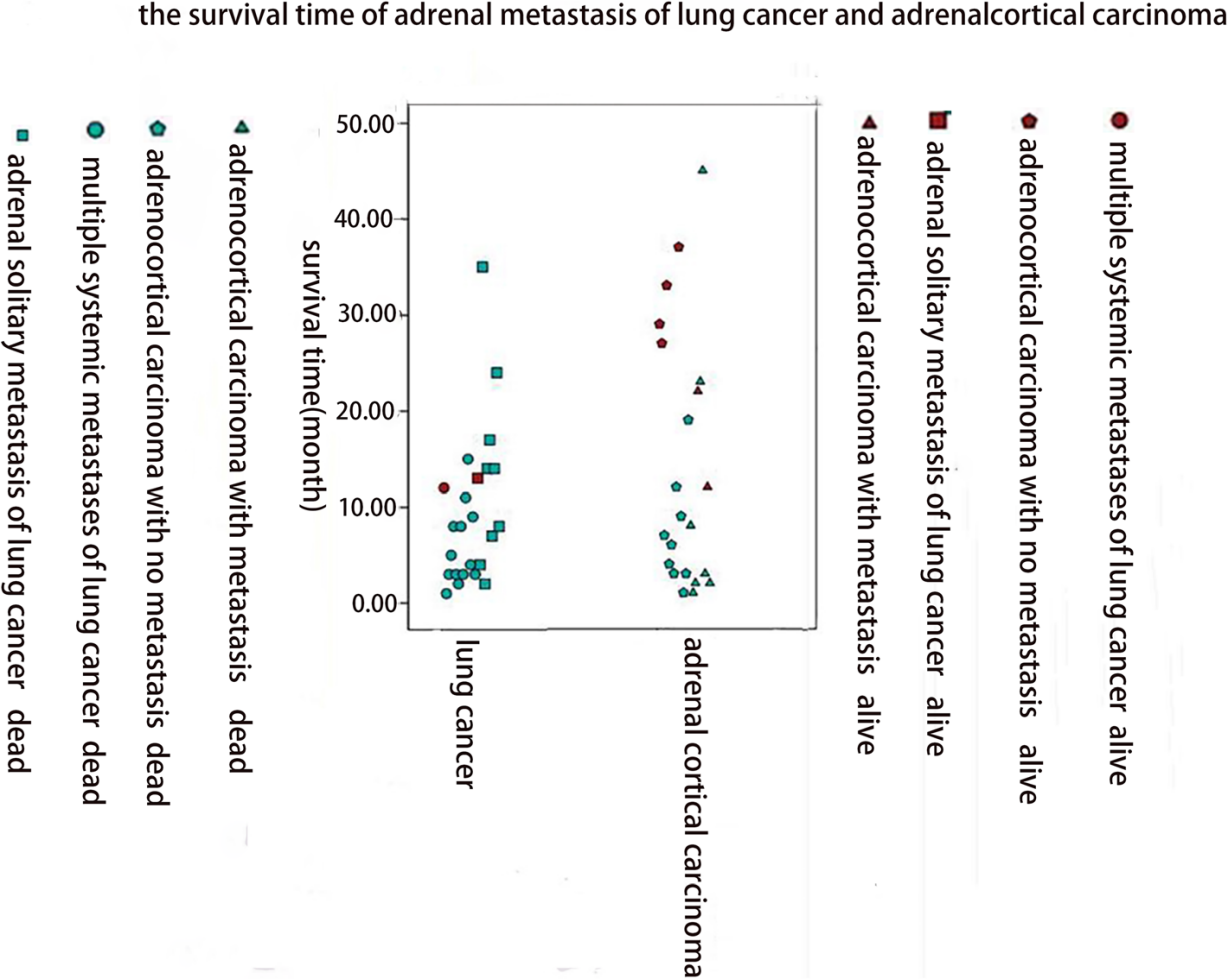
The survival time of adrenal metastasis of lung cancer and adrenal cortical carcinoma

**Fig. 3:**
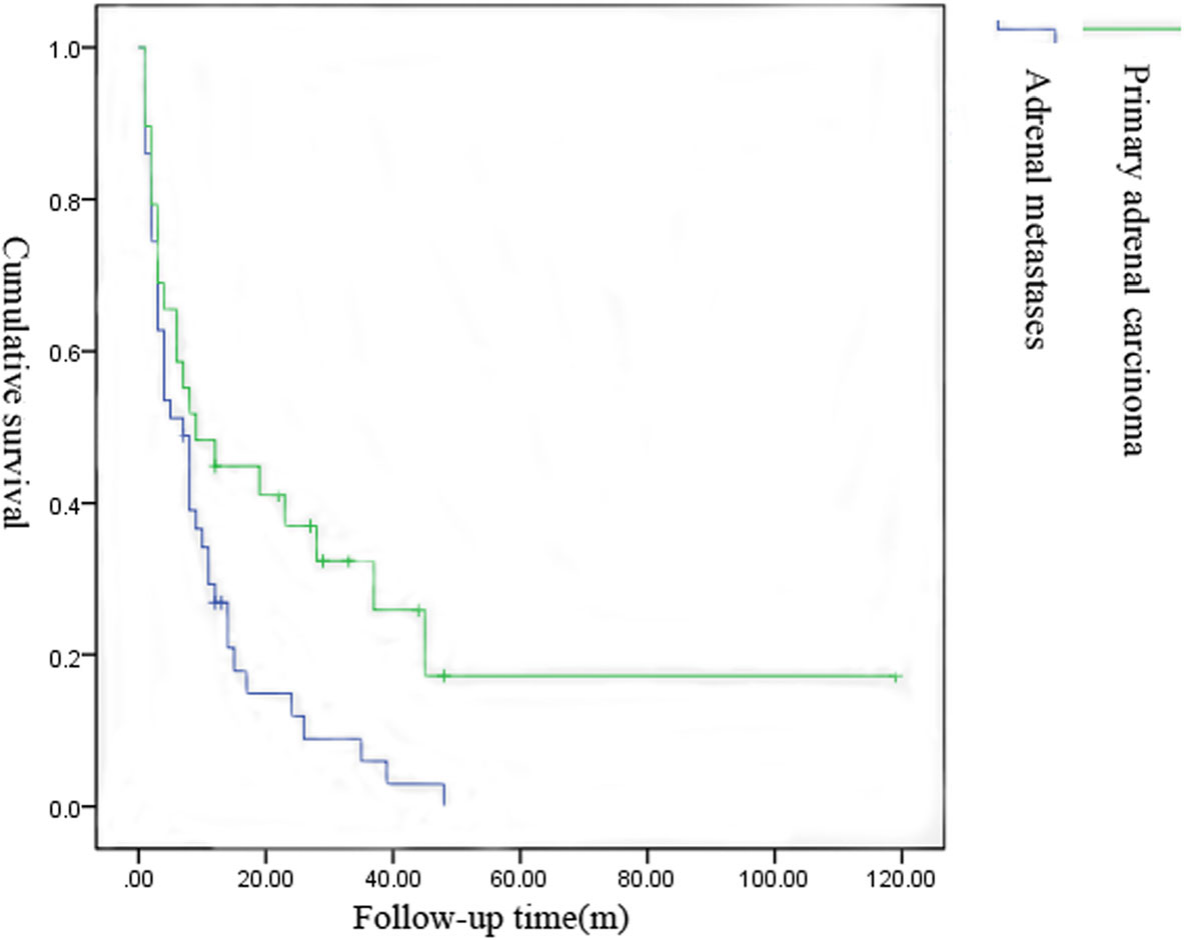
The survival graphs for adrenal metastases vs primary adrenal carcinoma

**Fig. 4:**
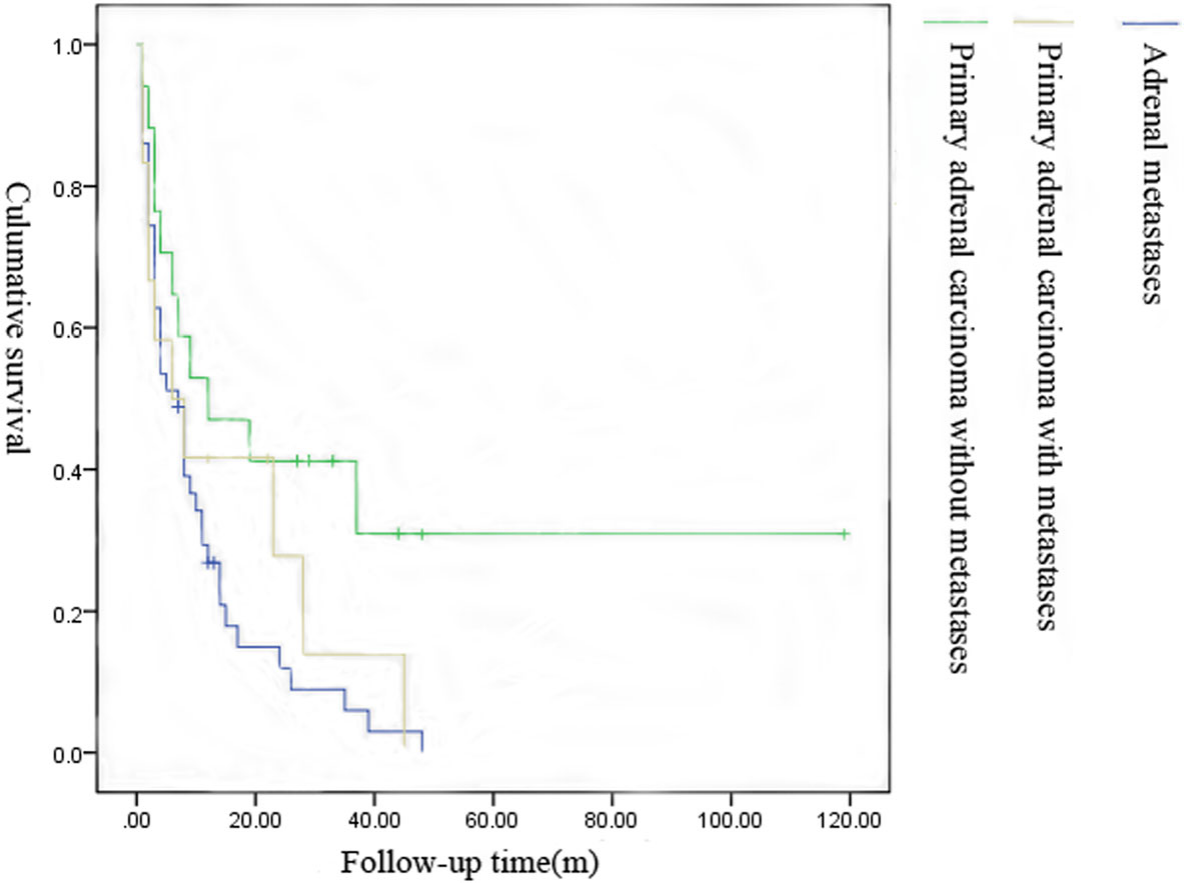
The survival graphs for adrenal metastases vs primary adrenal carcinoma without vs with metastases

Generally, the prognosis of adrenal malignant tumors was poor (Table 3). Lung cancer was the most common primary carcinoma of adrenal metastasis, of which 41.67% of cases were solitary adrenal metastasis and 58.33% were systemic multiple metastases including adrenal metastasis. The 2-, 6-, 12-, 24-, and 30-month survival rates of patients with lung cancer were 96%, 58%, 33%, 8%, and 4%, respectively. Adrenal cortical carcinoma accounted for 75.86% of the primary adrenal malignancy, 40.91% of which appeared distant metastatic at the time of initial diagnosis. The 2-, 6-, 12-, 24-, and 30-month survival rates of patients with primary adrenal cortical carcinoma were 91%, 64%, 45%, 23%, and 14%, respectively. In this cohort, the mean survival time of primary adrenal malignant tumors was 19.17 months and that of metastatic adrenal tumors was 9.49 months. In general, the prognosis of patients with primary adrenal carcinoma without metastases was relatively good (Figs. 1, 2, 3, and 4).

**Table 3:** The pathological distribution and survival of adrenal malignancy

Pathology types	Number	Survival rate (%)				
		2 m	6 m	12 m	24 m	30 m
Adrenal metastatic carcinoma	T (Mu/So)					
Lung cancer	24 (14/10)	96 (93/100)	58 (43/80)	33 (14/60)	8 (0/20)	4 (0/10)
Gastrointestinal cancer	5 (4/1)	60 (75/0)	20 (25/0)	0 (0/0)	0 (0/0)	0 (0/0)
Liver cancer	5 (1/4)	80 (0/100)	80 (0/100)	60 (0/75)	40 (0/50)	20 (0/25)
Renal carcinoma	5 (2/3)	80 (100/67)	60 (50/67)	20 (0/33)	20 (0/33)	20 (0/33)
Prostate cancer	1 (1/0)	0	0	0	0	0
Pancreatic cancer	1 (1/0)	100	0	0	0	0
Renal pelvis carcinoma	1 (1/0)	100	0	0	0	0
Nose malignant melanoma	1 (0/1)	100	0	0	0	0
Primary adrenal malignancy	T (N-Me/Me)					
Cortical carcinoma	22 (13/9)	91 (92/89)	64 (69/56)	45 (46/44)	23 (31/11)	14 (15/11)
Small cell carcinoma	3 (2/1)	100 (100/100)	100 (100/100)	100 (100/100)	100 (100/100)	67 (100/0)
Lymphoma	3 (2/1)	67 (100/0)	33 (50/0)	33 (50/0)	33 (50/0)	33 (50/0)
Melanoma	1 (0/1)	100	100	0	0	0

There was no significant difference between open adrenalectomy and laparoscopic adrenalectomy with respect to gender, age, or tumor location (Table 4). However, tumor diameter was larger in the open adrenalectomy group than in the laparoscopic adrenalectomy group (*P* < 0.05). There were more benign and nonfunctional LATs in the laparoscopic adrenalectomy group than in the open adrenalectomy group (*P* < 0.05). Finally, there were significant differences in intraoperative bleeding (251.8 > 63.09 ml), blood transfusion (202.25 > 8.99 ml), operation time (2.35 > 1.96 h), hospitalization time (20.36 > 14.72 days), and postoperative drainage time (5.58 > 3.75 days) between the two groups.

**Table 4:** The difference between open and laparoscopy

		Open	Laparoscopy	*P* value
Age (years)		52.21 ± 13.64	48.34 ± 15.98	*P* = 0.077
Size (cm)		8.37 ± 2.71	6.74 ± 2.10	*P* = 0.00
Gender	Male	38	42	*P* = 0.548
	Female	51	47	
Location	Right	46	39	*P* = 0.231
	Left	43	50	
	Unilateral	80	84	*P* = 0.267
	Bilateral	9	5	
Type	Benign	74	83	*P* = 0.037
	Malignant	15	6	
	Non-function	47	60	*P* = 0.047
	function	42	29	
Intraoperative	Bleeding	251.80 ± 503.28	63.09 ± 91.74	*P* = 0.00
	Transfusion (ml)	202.25 ± 378.64	8.99 ± 59.28	*P* = 0.00
	Injury	2	0	
Operative time (h)		2.35 ± 1.03	1.96 ± 0.88	*P* = 0.012
Conversion		6		
Postoperative	Fasting time (days)	3.03 ± 1.13	2.39 ± 0.84	*P* = 0.00
	Drainage time (days)	5.58 ± 2.72	3.75 ± 3.06	*P* = 0.00
	Hospital stay (days)	20.36 ± 8.07	14.72 ± 5.66	*P* = 0.00
Postoperative complication (Clavien)	I	46	62	
	II	27	21	
	III	0	0	
	IV	15	6	
	V	1	0	

CT features were compared among five types of LATs: benign adenomas, pheochromocytomas, cortical carcinomas, metastatic carcinomas, and gangliocytomas. As shown in Table 5, cortical carcinomas and gangliocytomas had a larger diameter. In terms of tumor shape, a large proportion of benign adenomas and pheochromocytomas were round (94.12%), while metastasis was more common in lobulated tumors (47.06%). Among the infiltrative tumors, there was a high proportion of cortical carcinomas (50%). Benign adenomas (97.06%), pheochromocytomas (94.29%), and gangliocytomas (85.71) were mostly circumscribed, while cortical carcinomas mostly presented ill-defined tumor margins (47.83%). Pheochromocytomas (94.29%), cortical carcinomas (88.89%), and metastatic carcinomas (95.65%) were mostly heterogeneous. However, the majority of gangliocytomas were homogeneous. Ninety-four percent of the pheochromocytomas were accompanied by necrosis, while most of the gangliocytomas had no necrosis. Tumors with calcification were more likely to be benign adenomas, whereas metastatic carcinomas and gangliocytomas had no calcification. CT features had readily visible specificity for the diagnosis of benign and malignant tumors; for example, benign adenomas had a smaller pre-contrast (< 10 Hu) which attenuation of other types of adrenal tumors did not have, whereas the contrast attenuation was more pronounced in pheochromocytoma than in other groups.

**Table 5:** CT characteristics of 117 lesions by clinical diagnosis

CT characteristics		Benign adenoma (*n* = 34)	Pheochromocytoma (*n* = 35)	Cortical carcinoma (*n* = 18)	Metastatic carcinoma (*n* = 23)	Gangliocytoma (*n* = 7)
Diameter (cm)		6.55 ± 1.76	7.6.1 ± 2.27	9.37 ± 3.46	7.53 ± 2.44	8.18 ± 2.76
Shape	Round	32 (94.12%)	30 (85.71%)	7 (38.89%)	13 (56.52%)	6 (85.71%)
	Lobulated	2 (5.88%)	2 (5.71%)	5 (27.78%)	8 (34.78%)	0
	Infiltrative	0	3 (8.57%)	6 (33.33%)	2 (8.70%)	1 (14.29%)
Margin	Circumscribed	33 (97.06%)	33 (94.29%)	7 (38.89%)	15 (65.22%)	6 (85.71%)
	Ill-defined	1 (2.94%)	2 (5.71%)	11 (61.11%)	8 (34.78%)	1 (14.29%)
Heterogeneity	Homogeneous	19 (55.88%)	2 (5.71%)	2 (11.11%)	1 (4.35%)	6 (85.71%)
	Heterogeneous	15 (44.12%)	33 (94.29%)	16 (88.89%)	22 (95.65%)	1 (14.29%)
Necrosis	Negative	20 (58.82%)	2 (5.71%)	6 (33.3%)	10 (43.48%)	6 (85.71%)
	Positive	14 (41.18%)	33 (94.29%)	12 (66.67%)	13 (56.52%)	1 (14.29%)
Calcifications		14 (41.18%)	6 (14.14%)	5 (27.78%)	0	0
Pre-contrast attenuation (HU)		23.94 ± 16.16	40.17 ± 7.78	36.89 ± 10.59	32.35 ± 9.05	32.86 ± 8.51
Contrast attenuation (HU)	Arterial phase	50.94 ± 23.01	87.46 ± 38.44	57.28 ± 17.67	55.26 ± 18.84	46.86 ± 28.29
	Venous phase	68.59 ± 31.95	91.17 ± 26.74	73.44 ± 29.22	58.13 ± 16.55	50.14 ± 24.12
	Delay phase	55.32 ± 26.32	79.37 ± 16.25	69.61 ± 24.75	53.48 ± 14.64	46.71 ± 17.81

## Discussion

Adrenal tumors are occasionally found in patients undergoing abdominal radiology, and the prevalence of such findings increased with increasing age [13, 14]. Generally, the larger the tumor, the greater possibility of adrenal cancer [3–6]; therefore, it was even recommended that a tumor size greater than 5 cm should be considered an additional criterion for surgical treatment of adrenal incidental tumors [15]. For this reason, we decided to analyze tumors of this size to provide experience and basis for preoperative differentiation and selection of appropriate treatment approaches of LATs.

LATs covered a wide range of pathological types. In other studies, benign LATs accounted for 62.59%, and malignant LATs accounted for 37.41% [3]. This is similar to our present study. Thus, in clinical diagnosis of LATs, the possibility of benign tumors should be considered first. However, a LAT still had a high probability of being a malignant tumor.

The presence of bilateral masses accounts for about 15% of the incidental adrenal tumors [16, 17]. In our study, except for malignant lesions, bilateral adrenal adenoma and lymphoma were the most common diagnoses. Metastatic or invasive neoplasms, congenital adrenal cortical hyperplasia, bilateral adrenal cortical adenomas, and ACTH-independent macronodular adrenal hyperplasia were described by certain authors as the most likely diagnosis. Others did not find a difference in the frequency of malignant lesions between patients with bilateral adrenal tumors and unilateral ones [17]. In our group, however, the malignant rate of bilateral adrenal tumor was higher (71.88% vs 22.83%). This difference was caused by the high proportion of metastatic carcinomas. In this way, bilateral LATs should be suspected of malignancy, and further examination should be performed to exclude metastases.

Imaging diagnosis is of great value in estimating the nature of LATs. CT, MRI, and FDG-PET/CT can be used in clinical diagnosis of LATs. We compared the imaging features of the following five common LATs: (1) cortical carcinoma: larger diameter, infiltrative, ill-defined tumor margin, heterogeneous; (2) metastatic carcinoma: lobulated tumors, heterogeneous, few calcifications; (3) benign adenomas: round, circumscribed, calcification; (4) gangliocytoma: larger diameter, circumscribed, homogeneous, no necrosis, few calcifications; and (5) pheochromocytoma: round, circumscribed, heterogeneous, necrosis, higher contrast attenuation.

Other authors described the presence of fat and a pre-contrast attenuation under 10 Hounsfield units have been found to be correlated with benignity [18, 19]. This is in accordance with our present study of LATs where benign adenomas had a pre-contrast attenuation (< 10 Hu) while other types of LATs did not have. Some scholars even thought that lesions with a pre-contrast attenuation of less than 10 Hu may be considered benign regardless of size [20]. However, in patients with adrenal metastases, 7% of cases with noncontrast HU ≤ 10 turned out to be malignant [21]. In our series, metastases had no calcification, which was similar to other studies except one [22]. This shows that CT cannot completely determine the nature of the tumor, and more comprehensive imaging examination was needed. In this case, contrast-enhanced washout CT was able to provide further information, showing that adenomas took up intravenous CT contrast rapidly and had a rapid loss of contrast, whereas malignant adrenal lesions usually became enhanced rapidly but had a slower loss of contrast [23, 24].

Other imaging methods, such as MRI and PET/CT, can further differentiate the benign and malignant adrenal tumors [25, 26]. PET/CT especially might be effective in finding the extra-adrenal malignancies with low uptake [27, 28].

Laparoscopic adrenalectomy is widely used in the world, and it is the first choice for small adrenal benign tumors, on account of its advantages, including reduced postoperative pain, shorter hospitalization and recovery times, reduced complication rates, and better cosmetic results [29]. These advantages of laparoscopic surgery may be related to the small tumor volume and greater number of benign tumors in this group. More prospective clinical studies are needed to compare the safety and efficacy of laparoscopic versus open surgery in the treatment of LATs. With the development of laparoscopic surgery and robotic surgery [30], tumor diameter has ceased to be a limiting factor in the surgical management of adrenal benign tumors. Laparoscopic adrenalectomy is preferred for benign LATs regardless of size or for LATs with radiological findings suspicious of malignancies and a relatively small diameter (according to the experience of the surgeon) but no evidence of local invasion. For malignant LATs, especially those with local invasion, open surgery might be a more reasonable choice. Thus, the selection of surgical method should be evaluated comprehensively according to the experience of the surgeon and the characteristics of the tumor.

Systemic imaging examination before LAT surgery and screening malignant lesions in other parts and selecting suitable treatment for patients were of great significance. Among adrenal malignant tumors, 59.72% were metastatic and 40.28% were primary. Among metastases, 55.81% were accompanied by multiple systemic metastases. At the time of diagnosis of primary adrenal carcinomas, 41.38% of patients had distant metastasis. From our results, both cases of primary adrenal cancer and adrenal metastasis, once accompanied by metastases in other parts of the body, showed poor prognosis. In this case, surgical treatment was not the most appropriate method. In other studies, adrenocortical carcinoma patients had a very poor prognosis with a 5-year overall survival below 30% in most series [31]. Age, tumor property, general health, comorbidities, and patient preference should be taken into account in order to maximize the benefits to patients.

Our research had some specific limitations. The number of cases in our single center was limited, and there were only a few tumors of rare pathology. There may be a large bias in the evaluation of clinical features and survival prognosis of these patients; for some patients with adrenal metastases, the diagnosis mainly depended on clinical diagnosis, without any confirmation from pathological diagnosis. In some patients with bilateral tumors, pathology was only performed on one side of the body, which may also affect the statistical results of pathological distribution in this group of cases.

## Conclusion

LATs were here affirmed to be a group of complex diseases with diverse sources. Understanding the clinicopathological characteristics of LATs was found to be of great significance for accurately evaluating LATs and selecting the most suitable treatment.

## Data Availability

The data came from our hospital’s data system.

## Abbreviation

LATs: Large adrenal tumors
